# Transcriptome walking: a laboratory-oriented GUI-based approach to mRNA identification from deep-sequenced data

**DOI:** 10.1186/1756-0500-5-673

**Published:** 2012-12-05

**Authors:** Andrew S French

**Affiliations:** 1 Department of Physiology and Biophysics, Dalhousie University, PO BOX 15000, Halifax, NS B3H 4R2, Canada

**Keywords:** Deep-sequencing, Assembly, GUI, Transcriptome, Bioinformatics

## Abstract

**Background:**

Deep sequencing technology provides efficient and economical production of large numbers of randomly positioned, relatively short, estimates of base identities in DNA molecules. Application of this technology to mRNA samples allows rapid examination of the molecular genetic environment in individual cells or tissues, the transcriptome. However, assembly of such short sequences into complete mRNA creates a challenge that limits the usefulness of the technology, particularly when no, or limited, genomic data is available. Several approaches to this problem have been developed, but there is still no general method to rapidly obtain an mRNA sequence from deep sequence data when a specific molecule, or family of molecules, are of interest. A frequent requirement is to identify specific mRNA molecules from tissues that are being investigated by methods such as electrophysiology, immunocytology and pharmacology. To be widely useful, any approach must be relatively simple to use in the laboratory by operators without extensive statistical or bioinformatics knowledge, and with readily available hardware.

**Findings:**

An approach was developed that allows *de novo* assembly of individual mRNA sequences in two linked stages: sequence discovery and sequence completion. Both stages rely on computer assisted, Graphical User Interface (GUI)-guided, user interaction with the data, but proceed relatively efficiently once discovery is complete. The method grows a discovered sequence by repeated passes through the complete raw data in a series of steps, and is hence termed ‘transcriptome walking’. All of the operations required for transcriptome analysis are combined in one program that presents a relatively simple user interface and runs on a standard desktop, or laptop computer, but takes advantage of multi-core processors, when available. Complete mRNA sequence identifications usually require less than 24 hours. This approach has already identified previously unknown mRNA sequences in two animal species that currently lack any significant genome or transcriptome data.

**Conclusions:**

As deep sequencing data becomes more widely available, accessible methods for extracting useful sequence information in the biological or medical laboratory will be of increasing importance. The approach described here does not rely on detailed knowledge of bioinformatic algorithms, and allows users with basic knowledge of molecular biology and standard laboratory computing equipment, but limited software or bioinformatics experience, to extract complete gene sequences from deep-sequencing data.

## Findings

### Availability and requirements

**Project homepage [**[1]**]:**http://asf-pht.medicine.dal.ca/Downloads/

**Operating system:** Windows (64-bit only).

**Programming language:** Visual C++, Microsoft Visual Studio.

**Other requirement:** 64-bit Intel processor (or equivalent), 4 GB memory.

**License:** Compiled installer package is freely available and provided as Additional file 1, [2].

**Data:** Two files containing data that can be used to operate the program and perform the complete walk illustrated in the manuscript are available as Additional files 2 and 3.

### Introduction

Technology for DNA sequencing is developing rapidly, and sequencing of cDNA derived from cell or tissue RNA (RNA-Seq) allows relatively easy access to the transcribed RNA, or transcriptome, of almost any tissue [3-6]. This opens many new opportunities for laboratories that have traditionally relied on functional or morphological techniques, to obtain mRNA data for the tissues under investigation. This, in turn, can help to solve problems that require detailed molecular structures of cellular proteins and their products.

While acquisition of genomic and transcriptome data becomes easier and more affordable, methods for processing the sequence data to obtain complete DNA or RNA sequences have not developed at the same pace [7,8]. At the time of writing, a typical sequencing run by the Illumina process, for example, produces >10^8^ sequences, or ‘reads’ of ~10^2^ base length, randomly positioned to the original molecules (‘reads’ are similar to short sequences of cDNA produced by earlier sequencing technology, commonly called Expressed Sequence Tags or ESTs). This amount of data presents significant analysis and processing problems. When complete genomic data is available, it is possible to search for known or putative sequences, but in the absence of such information, some form of *de novo* assembly is required. Complete *de novo* assembly of 10^8^ reads by searching for overlaps between reads would require years by a single processor, leading to the development of alternative approaches. Curiously, many of these have relied on reducing the data to even shorter, fixed-length sequences, sometimes called *k-mers*, before constructing de Bruijn graphs [6,8,9]. However, while the field is progressing, the existence of many competing approaches, and the complexity of measuring effectiveness, indicates that *de novo* assembly is still immature, for both genomic and transcriptomic data [6,7].

The present work grew out of a need in our own laboratory for detailed molecular structures of several specific proteins involved in sensory transduction and its modulation in a spider sensory organ [10]. While it was relatively easy to obtain the raw data of the transcriptome for the tissue, discovery and assembly of the specific sequences of interest was not easily available because of the absence of genomic information for this, or any other spider. The method that we developed attempts to deal with the large amount of data, while simultaneously allowing the operator to directly view as much information about the data as possible. In particular, we attempted to make the large number of reads, and the extensive overlapping of the reads, into advantages, rather than disadvantages. The method is designed for the task of identifying specific individual mRNA sequences, rather than a complete collection of transcribed genes. The presentation here used data from Illumina RNA-Seq operations, but is sufficiently general to be applied to other sequence data.

The software described here provides a single program (Additional file 1) for extracting complete mRNA sequences from commercial RNA-Seq data that can be used by operators with knowledge of basic molecular biology but without detailed knowledge of bioinformatics. The program runs on a standard desktop or laptop computer and provides an easily understood graphical model of the data being processed at each stage.

### RNA-Seq data

Tropical wandering spiders, *Cupiennius salei* were maintained in a laboratory colony at room temperature (22 ± 2°C) and a 13:11 h light:dark cycle. Eight legs from an adult male spider were autotomized following a protocol approved by the Dalhousie University Committee on Laboratory Animals. Total RNA (81 μg) was extracted from the combined legs using a Qiagen RNeasy plus universal midi kit and following the manufacturer’s instructions. Separation of mRNA, construction of cDNA library and Illumina processing were performed by McGill University and Génome Québec Innovation Centre, Montréal, Québec. The cDNA fragments had an average length of 219 ± 50 bases. Illumina processing gave paired reads of 100 bases commencing from either end of each fragment. These will be referred to as primary reads and their paired ends. The raw data consisted of 89,919,581 such pairs of 100 base reads with associated quality values (phred values) in Illumina Casava 1.8 structured files. The primary reads and paired ends were in two separate files. The raw data were groomed to remove any sequence containing less than 80 contiguous bases with Phred score > 19 (probability of error in base identification less than 1%), to yield files of 60,110,040 and 70,141,080 reads of 80–100 bases.

All operations on the data were performed within one computer program, written in Visual C++ using Microsoft Visual Studio (Additional file 1). Data within the program was organized into software structures termed DataSets, with each DataSet containing one or more Sequence software structures. Each Sequence structure contained the sequence itself, matching Phred quality values, and additional information defining its alignment to a target sequence.

Groomed data was held in standard disk files in Casava 1.8 structured files. Visual C++ allows programming of multiple parallel software tasks, termed ‘threads’. If multiple processors are available, threads can run in parallel, increasing the data processing efficiency. Searching and walking operations were performed on both data files simultaneously using a triple-thread organization. Two identical threads performed the actual searches on the primary reads and paired ends, transmitting their results to a supervisory thread that performed all executive functions and produced the user display.

### Searching for specific sequences

Targets for searches were selected from fruit fly, *Drosophila melanogaster* ( http://www.ncbi.nlm.nih.gov, http://www.flybase.org) or brown dog tick, *Rhipicephalus sanguineus*, (Lees et al. 2010) sequences. Typically, 200 base segments from highly conserved regions were searched at low stringency (15–20 contiguous base matches). Searches were performed on the two files containing paired ends simultaneously, using the triple-thread method.

**Figure 1:**
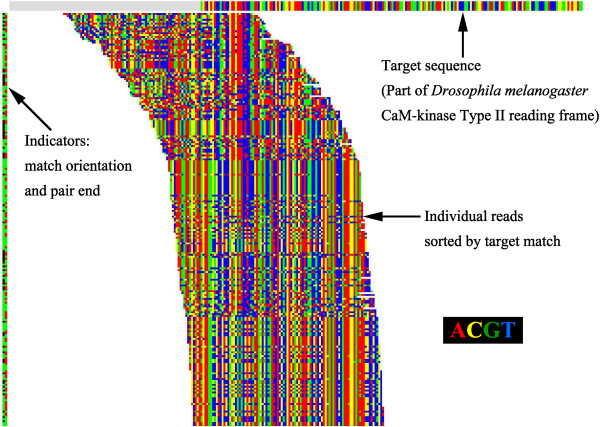
**Discovering putative matches to an initial target sequence (part of *Drosophila *CaM-kinase, [GenBank: BT050453.1]).** All bases are shown as colored blocks, using the indicated color code. The target sequence is above, with grey color indicating regions not used in the search. Each matched read is shown as a single horizontal line, located horizontally by its match position to the target sequence. Indicator color blocks at left show match orientation (normal - black, reverse complement - green) and pair end (primary - green, paired end - red). Less than half the 1,387 matching reads are shown. The reads are shown sorted into ascending match position to the target, but sorting by several other criteria, including number of matched bases, maximum identical base matches, etc. is possible.

An example of a typical search for calcium-calmodulin dependent protein kinase (CaM-kinase) is shown in Figure 1. The initial search was conducted against a *Drosophila* sequence [GenBank: BT050453.1]. This figure also illustrates the graphical user interface that was used for all major operations on the data. Sequences were displayed as color coded pixel blocks arranged in horizontal lines, with the target sequence at the top of the display. All base positions were numbered relative to the 5’-end of the target sequence, commencing at the left side of the display. The vertical order of sequences could be decided by a range of sorting options. In this figure the read sequences were sorted by their matching position to the target. Note that the figure contains a mixture of sequences from both of the Illumina paired end files, and that both direct and reverse complement matches were included, indicated by color-coded bars at left. However, each matched sequence now included a description of its alignment to the target sequence that allowed the program to display the correct match alignment. This alignment data stayed with the sequence as it progressed through further stages of processing, and was used by many of the other possible operations that the program provides (Table 1).

**Table 1:** Major operations provided by the software package

	
Operations on entire DataSets:	Opening and saving disk files in FASTA or FASTQ (Casava 1.8) format
	Grooming disk files based on quality scores
	Removing duplicate sequences
	Removing non-coding sequences
	Sorting sequence order by: pair end, sequence length, position of match to target sequence
Operations on selected sequences:	Changing sequence length by addition or subtraction of bases
	Shifting match position relative to a target sequence
	Translation of sequence to amino acids
	Moving sequences between DataSets
	Editing sequence identity codes or descriptions
Operations for sequence discovery and assembly:	Locating matching sequences in DataSets or disk files
	Viewing sequence matches to a target sequence
	Walking along a sequence by searching disk files
	Finding missing paired ends and matching them to the target sequence
	Melding matched sequences into a single sequence
	Assembling sequences by simple overlap detection

Several alternate further steps were available at this stage. The number of matching reads would usually be small enough to allow a complete assembly by sequence overlaps, provided within the program. A BLASTX search for sequences of interest could also be conducted. The program also allows the user to sort the individual sequences into overlapping sets by manual operation (mouse clicks), which may be useful when particular features are sought. It is also possible to automatically exclude sequences that do not contain a complete coding frame, assuming that the target sequence was within the reading frame.

The example here (Figure 1) gave 1,387 matching reads, of which 103 were immediately rejected as non-coding. Direct, user guided assembly of these yielded a set of 84 reads with perfectly overlapping regions of 21–79 bases, leading to an initial sequence of 346 bases that gave close BLAST matches to a range of arthropod CaM-kinase Type II mRNAs. This initial target sequence was selected for complete exploration.

The search for CaM-kinase also produced 29 other contiguous sequence fragments that were identified as various kinases. Together with similar searches for two types of ligand-activated ion channels and a metabotropic transmitter receptor we have so far identified a total of >250 contiguous fragments that are ready for the next stage of processing, walking.

### Walking

**Figure 2:**
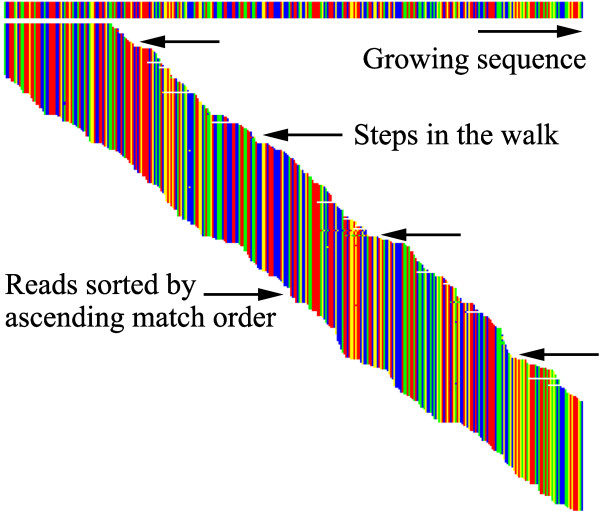
**Walking along the growing sequence.** The growing CaM-kinase sequence is shown using a similar display to Figure 1 during a walk towards the 3’ end. However, the upper target sequence is now the melded sequence from all of the discovered reads. Individual steps in the walk correspond to repeated complete searches through both paired end files, following which the newly discovered matching sequences are melded onto the end of the growing sequence. Several errors in sequencing and matching are evident, but the large numbers of overlapping reads allow quality-based voting in the melding process.

Typical steps in walking along a sequence are shown in Table 2. Once the initial sequence had been discovered it was possible to search through the raw data for overlapping reads at either end. This was performed at much higher stringency than the initial search to avoid spurious assembly. The program performs this operation automatically once the initial parameters are set (Table 2). These include the direction of the walk, the minimum number of overlapping bases required and whether any base errors are tolerated. The complete groomed data set is then searched for overlapping reads, which are each coded for alignment to the growing sequence as they are found, exactly as in the discovery process. This allows the same display screen to show the steps of the walk (Figure 2).

**Table 2:** Steps in performing a sequence walk

1.	Open the file containing the initial target sequence (usually one or more melded reads).
2.	Select direction of walk (it is operationally simpler to walk 3’ to 5’ first because this does not require later shifting the matching index of any previously matched reads).
3.	Select the target length, minimum base overlap (typically >30), maximum permissible errors per match (typically 0–2), minimum number of bases to meld a ragged end (typically >4), maximum number of reads to find per step (typically 50) and maximum number of reads to add per step (if less than the maximum to be found).
4.	Select the base orientations to test (depends on the data available - forward and reverse complement for an Illumina paired-end set).
5.	Click ‘Walk’. The program will ask for one or two data files to search, and then proceed.
6.	The program will finish walking when the data is exhausted (no more matching reads found) or the operator clicks ‘Stop’. At this point, any duplicate sequences can be removed (menu) and the data files can be searched for the missing member of any incomplete pair (menu). Any extra paired reads found can be matched to the main sequence (menu).
	At the end of a 3’ to 5’ walk the dialog will indicate the size of the origin shift (in bases). The matching sequences from any previous walk can then be shifted by that amount (menu). All matching reads (from both walks and any additional paired ends) can be combined into one set (menu). The combined set can then be melded to give the complete discovered sequence (menu).

The example in Figure 2 shows the CaM-kinase sequence (Additional file 2) extended towards the 3’ end in a series of steps. A minimum overlap of 30 bases in this case gave maximum steps of 70 bases, but overlap was often greater, leading to shorter, but more reliable, steps. Variation in the overlap matches give ragged ends to the steps. These ragged ends require another parameter to decide how many reads must contribute to the growing end of a step. In this example a minimum count of five reads was used. At the end of each step search the overlapping reads were melded onto the end of the growing sequence. The melding routine used a weighted vote for each additional base, with weight proportional to the quality (Phred score) value of the base in each read (Additional file 2).

Although the overlapping reads were melded, they were also retained in the growing DataSet, with their matching information, so that the user could see every read used in assembling the growing sequence. To minimize interference with the walking process, the complete DataSet, with new overlaps and the melded growing sequence, were automatically saved to a disk file at the end of each step. This allowed the user to inspect the process using a separate copy of the program. If errors were detected during this process (typically due to small numbers of reads at one or more points in the walk) the user could halt the main program and remove individual reads before re-melding and restarting the walk. In practice, such interventions were rarely required when the raw data was of good quality. Usually, the large numbers of reads available at each point along the sequence prevented occasional errors from disrupting the walk (a few examples of sequencing errors are evident in Figure 2). The process thus took advantage of the deep sequencing to reduce assembly errors and give the user a visual indication of assembly reliability.

Using a standard 3 GHz i7 desktop computer the walking process for the CaM-kinase gene took about 20 minutes per step, with a step size of about 60 bases, giving an overall growth rate of approximately 200 bases/hour. Since an i7 computer has eight effective processors, it was possible to run two versions of the program (using six of the processors) in opposite directions along the sequence, giving an effective rate of ~400 bases/hour. The complete reading frame of 1440 bases (Additional file 1) was therefore completed in less than four hours. It has also proved completely practical to run the program overnight on an i3 laptop computer.

### Duplicates and Paired ends

**Figure 3:**
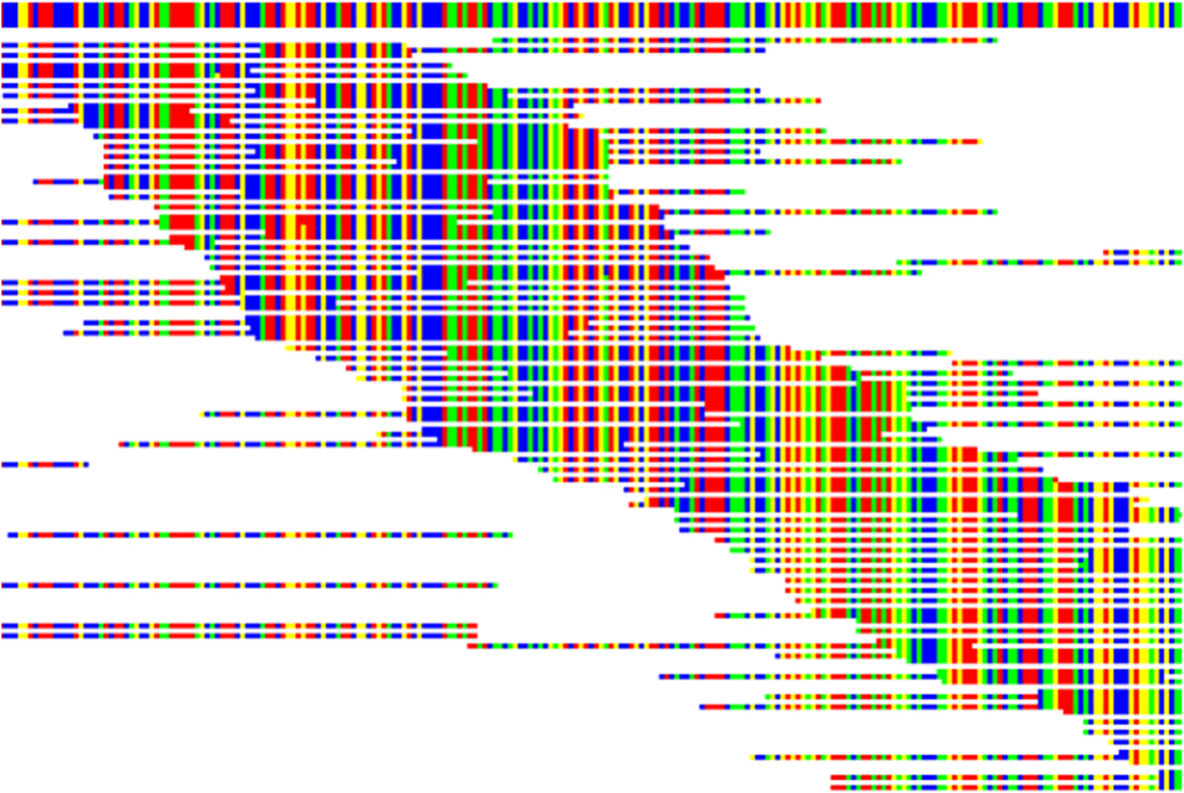
**Paired ends effectively increase read length.** A similar CaM-kinase walk display to Figure 2, but now the reads have been sorted to bring paired ends together, while retaining the match position to the growing sequence for one of each pair. Each read is still positioned horizontally according to its match position. The sorting algorithm also calculated the effective fragment length for each pair and gave estimates of the mean and range of fragment lengths.

Duplicate reads can enter the process if any steps of the walk are unusually short. At completion of walking the complete DataSet was checked for duplicate sequences, based on the identity codes of the reads, and duplicates were removed. The Illumina process currently produces reads of ~100 bases, but the paired ends of each cDNA fragment extends the effective read size to approximate the mean fragment size [11]. Although both members of many pairs are routinely captured in the walking process, our experience is that about one third of the pairings are normally incomplete. The program therefore provides a final search through the entire groomed data set to capture the missing pairs, using their sequence identities. The missing paired ends were then matched to their correct positions along the final sequence using the same routine as for initial sequence discovery. The program provides a sorting function that brings all the pairs together in order, while maintaining the order of the previous sorting for one of each pair (Figure 3). This function also calculates the effective size of each fragment from the difference in matched positions of the two ends. For the CaM-kinase data the fragment size range was 100–835 bases, with a mean of 196 bases. These values can be compared with the initial bioanalyzer estimate of 219 ± 50 for all fragmented cDNA.

### Depth of coverage and mutations

**Figure 4:**
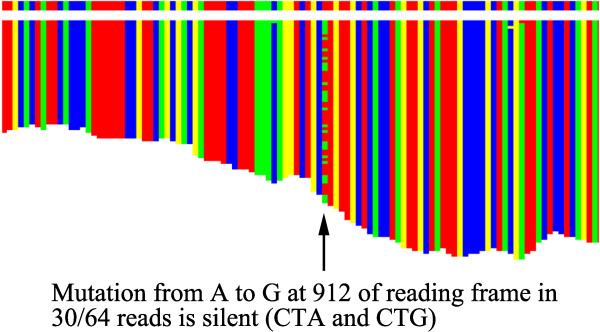
**Histogram display of matched reads.** The same CaM-kinase display converted into a histogram of base counts by collapsing the display onto the X-axis (melded sequence). This alternative display gives a better visual indication of the number of reads contributing to each base decision, and hence reliability. In this case, it also illustrates that two alleles are present in the reads.

An optional rearrangement of the colored pixels in the displays produces a histogram of the number of reads contributing to each base in the final sequence (Figure 4). This function also provides a visual image of the number of mutations or sequencing errors occurring at each position. For the CaM-kinase data the number of errors was small, but two alleles of the gene were immediately evident with a mutation from A to G in 30/64 reads at location 912 of the reading frame (Figure 4), giving a silent mutation of CTA to CTG.

### Resulting amino-acid sequence

**Figure 5:**
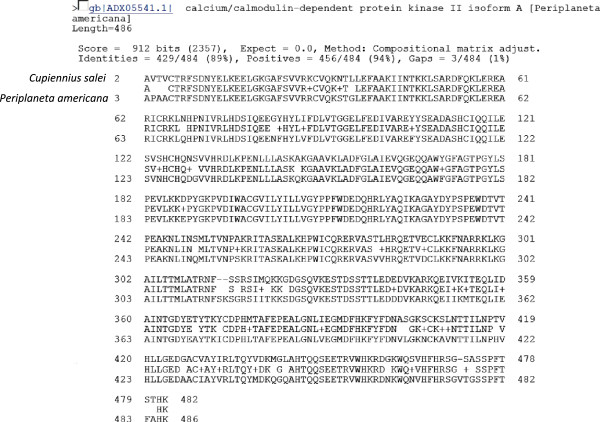
**Amino acid sequence comparison of the translated CaM-kinase reading frame by protein-protein BLAST against the non-redundant protein database.** The closest match was to the calcium/calmodulin-dependent protein kinase II isoform A of the American cockroach, *Periplaneta americana*. Other details are indicated in the figure.

The complete reading frame of the CaM-kinase data was translated by the program and entered into the protein-protein BLAST against the non-redundant protein database. The closest match (89% identical) was to calcium/calmodulin-dependent protein kinase II, isoform A, of *Periplaneta americana*, the American cockroach (Figure 5).

### Conclusions

Our initial objective was to identify the amino acid sequences of a series of molecules whose existence in *Cupiennius salei* was indicated by electrophysiological and immunocytochemical studies [2,12]. It is important to note that these requirements were significantly different to those of researchers interested in comparative genomics, or seeking quantitative data on gene translation in different tissues or conditions. However, our requirements are common to many studies that seek to understand physiological processes, and the proteins involved at each stage of those processes e.g. [3]. Following RNA separation from *Cupennius salei*, and Illumina sequencing, we found that very limited resources were available for specific gene identification from the data because of the lack of genomic data. We needed a relatively simple and straightforward method of finding mRNA sequences from the deep sequencing data that could be used by experimental laboratory members without extensive bioinformatics training, and using standard laboratory desktop or laptop computers.

The method that we have developed allows the user to perform all the major steps in finding and completing a transcribed gene using a single program [2] on a laboratory desktop computer. The only knowledge required is a basic understanding of mRNA transcription and translation, combined with reasonable estimates of the number of overlapping bases that should be expected from reads that originate from the same fragmentary sequence, or that come from genes with close homology to similar genes in a related species that can provide a template.

The single mutation in the CaM-kinase reading frame (Figure 4) illustrates an important limitation of sequencing methods that produce short reads. While no other mutations were seen in this CaM-kinase reading frame, other mutations were present in the 3’ noncoding region, and other *Cupiennius* genes that we have explored had multiple mutations within the reading frame. If such mutations are further apart than the average cDNA fragment length it becomes impossible to decide which mutations are associated with each other in the alleles that produced the original data.

As described above, the program provides a method of finding an initial set of reads from the mRNA of interest. However, other approaches and information, such as genomic data if available, could also be used for this step. The major development here is in the second stage, walking, where the numerous overlapping reads provided by deep sequencing allow an easily comprehended, but highly reliable and efficient method of completing de novo synthesis of the complete sequence. In addition, the graphical user display (Figures 1, 2, 3 and 4) provides a crucial resource by giving the user an immediate and accessible understanding of the data being processed and its internal relationships. Using standard laboratory computers, a transcribed gene can usually be discovered and sequenced reliably in a period of a few hours to one day, which is a very acceptable time period for most functional studies.

## Competing interests

The author declares that he has no competing interests.

## Supplementary Material

Additional file 1 Sequence.exe Self-unzipping executable Windows installer package of the program. Click here for file

Additional file 2 Cupiennius_0162a.fastq Nucleotide sequence of the CaM-kinase reading frame. Click here for file

Additional file 3 **Cupiennius_0162b.fastq Set of nucleotide sequences that generated the reading frame.** The two nucleotide files are in FASTQ format with Casava 1.8 quality score coding. Sequences in the Cupiennius_0162b.fastq file contain matching information to the reading frame. They can be viewed against the reading frame directly by opening them in the Sequence program. Click here for file
